# Pediatric unit spending in the North of Italy during the COVID-19 pandemic

**DOI:** 10.1186/s13052-023-01486-9

**Published:** 2023-07-13

**Authors:** Roberto Franceschi, Evelina Maines, Angelamaria Petrone, Simone Bilato, Ilaria Trentini, Lorenzo Di Spazio, Luca Leonardi, Massimo Soffiati, Andrea Francesconi

**Affiliations:** 1 Pediatric Unit, S.Chiara Hospital of Trento, APSS, Trento, Italy; 2 Planning and management control Service, Azienda Provinciale per i Servizi Sanitari, APSS, Trento, Italy; 3 Hospital Pharmacy Department, S. Chiara Hospital of Trento, Trento, Italy; 4 Drug policy service and pharmaceutical assistance, Azienda Provinciale per i Servizi Sanitari, APSS, Trento, Italy; 5 Department of Economics and Management, University of Trento, Trento, Italy

**Keywords:** Pediatric Unit, Budget, Spending, Cost, COVID-19

## Abstract

**Background:**

During the COVID-19 pandemic, accesses to pediatric health care services decreased, as well as the consumption of traditional drugs, while the median cost per patient at the emergency department slightly increased and the cost of pediatric COVID-19 admissions to the pediatric ward too. Overall spending of a secondary level Pediatric Unit in the last two years has not been previously reported.

**Methods:**

This is a retrospective study conducted by the Pediatric Unit of S. Chiara Hospital of Trento, North of Italy. We collected data on consumption and spending before and during the COVID-19 pandemic (between January 2018 and December 2022).

**Results:**

The total spending ranged from 2.141.220 to 2.483.931 euros between 2018 and 2022. COVID-19 spending accounted only for 5–8% of the overall budget, while two macro-areas of spending were identified: (i) biologic drugs for inherited metabolic diseases (IMDs), that impacted for 35.4–41.3%, and (ii) technology devices for type 1 diabetes (T1D), that accounted for 41.6–32.8% of the overall budget, in 2021 and 2022, respectively. Analysis of costs along with the different health care services revealed that: (i) the spending for COVID-19 antigen tests and personal protective equipment had a major impact on the Emergency room budget (from 54 to 68% in the two years); (ii) biological drugs accounted mainly on the Pediatric Ward (for 57%), Day Hospital (for 74%) and rare disease center budget (for 95% of the spending); (iii) the cost for T1D devices was mainly due to continuous glucose monitoring, and impacted for the 97% of the outpatient clinic budget.

**Conclusions:**

The main impact on the budget was not due to COVID-19 pandemic related costs, but to the costs for biologic drugs and T1D devices. Therefore, cost savings could be mainly achieved through generic and biosimilars introduction and with inter-regionals calls for technology devices. We emphasize how the control of spending in pediatric hospital care has probably moved from the bedside (savings on traditional drugs as antibiotics) to the bench of national or inter-regional round tables, to obtain discounts on the costs of biologic drugs and medical devices. Here we provide for the first-time in literature, data for bench-marking between secondary level Pediatric Units before and during the COVID-19 pandemic.

## Background

The pediatric health care system in Italy is made up of three main levels of intervention: first access/primary care, secondary care/hospital care, and tertiary care based on specialty hospital care [1]. A key focus of health policies in recent decades was to promote a shift away from unnecessary inpatient care, and in response total pediatric beds in hospitals decreased significantly during the last 15 years [2].

In this scenario, Coronavirus disease 2019 (COVID-19) caused by severe acute respiratory syndrome coronavirus 2 (SARS-CoV- 2), was declared a pandemic by the World Health Organization, and the impact was not only in terms of acute and long-term health consequences, but also on social and economic outcomes. There is a paucity of studies examining the impacts of the pandemic on secondary care hospital budgets: previous studies that estimated in-patient health care costs directly associated with an acute COVID-19 in adults, found that budget impact was greater than multiple sclerosis, cancer and diabetes cost [3], and drug expenditures were consistently altered [3–6].

In children, COVID-19 caused mild disease and rarely had a serious presentation. Starting from 2020, the COVID-19 pandemic and the implementation of mitigating measures caused a significant reduction in common seasonal pediatric viral and bacterial infectious diseases disseminated through droplets and contact, while other conditions as accidents, ingestions, neurological and eating behavior disorders increased [7–9]. In parallel, a significant reduction in the annual expenditure for pediatric antibiotics, salbutamol and inhaled corticosteroids was observed [10, 11].

As a consequence of reduced infectious, ward admissions, outpatient visits, and emergency room access in pediatric settings [10] decreased, while telemedicine use has become a new structured reality for the follow up of chronic diseases [12, 13]. Overall costs at the pediatric emergency department during the pandemic period have been reported as reduced, while median cost per patient slightly increased from 2019 to 2020 [7] and direct median costs among cases of pediatric COVID-19 were increased 1,7 − 1,8 times compared with controls [14].

The objective of this study was to analyze the impact of COVID-19 pandemic on the spending of a secondary level Pediatric Unit of the North of Italy during the last two years, as this data has not previously been reported and in the pediatric subjects the COVID-19 impact was different from that on the adults. Knowledge of trends in pharmaceutical, medical devices and other products spending, could support healthcare leaders in planning future expenses for their organizations.

## Methods

This is an observational retrospective study conducted by the Pediatric Unit of S. Chiara Hospital of Trento, North of Italy.

### Pediatric Unit organization and performance volumes

In our country, the Autonomous Province of Trento, subjects aged 0–14 years were 75.267 in 2021, and health care is provided free of charge by the national health service (NHS). In our Province, there is only one company that provides public hospital and territorial health care (Azienda Provinciale per i Servizi Sanitari – APSS) and general hospitals are organized on a hub and spoke care delivery model. The S. Chiara Hospital of Trento includes secondary level Pediatric Services, and works collaboratively with spoke hospitals (Rovereto and Cles) that include pediatric primary level of care.

**Fig. 1:**
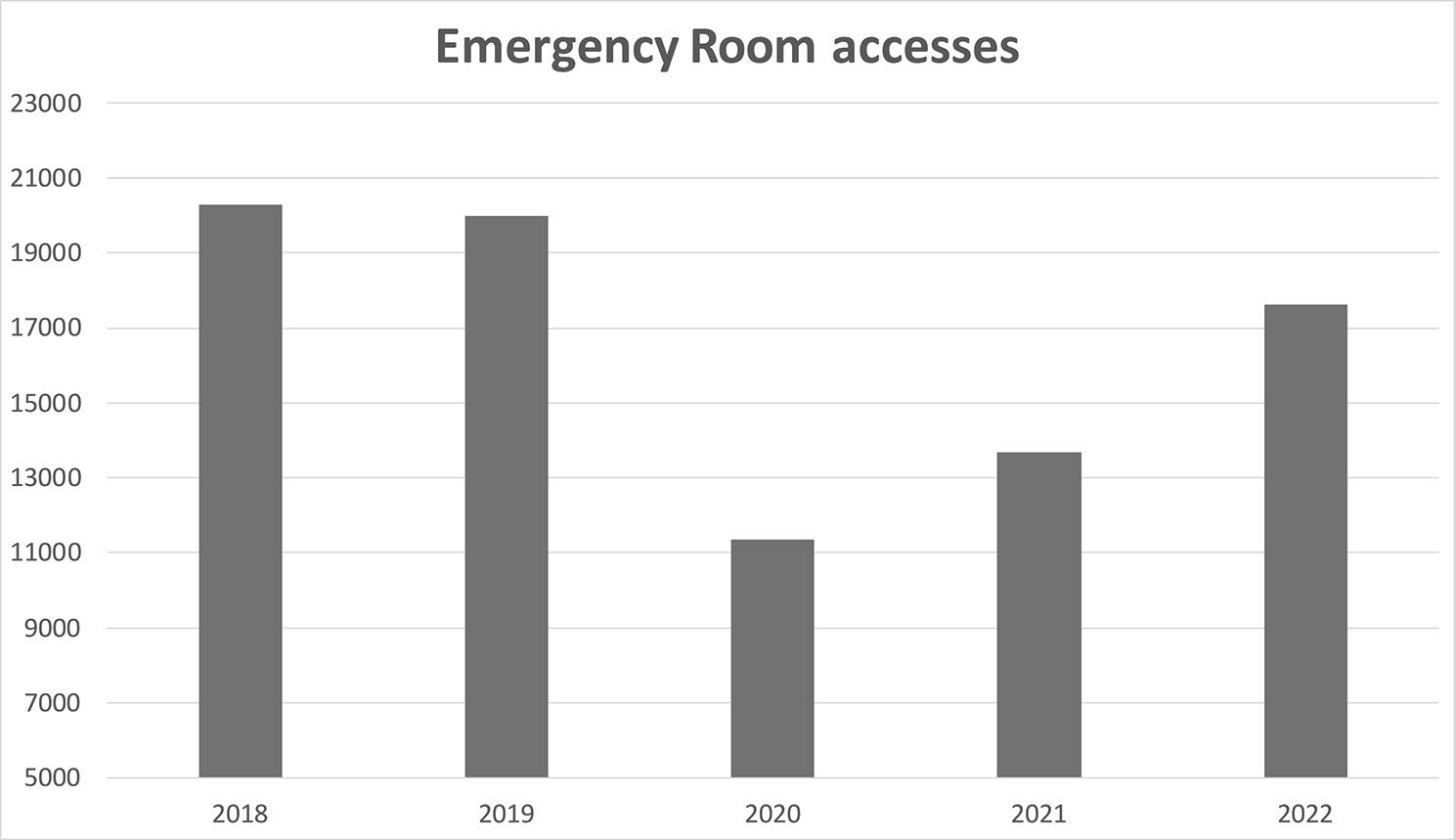
Volumes of accesses in the last few years at the Emergency room

**Fig. 2:**
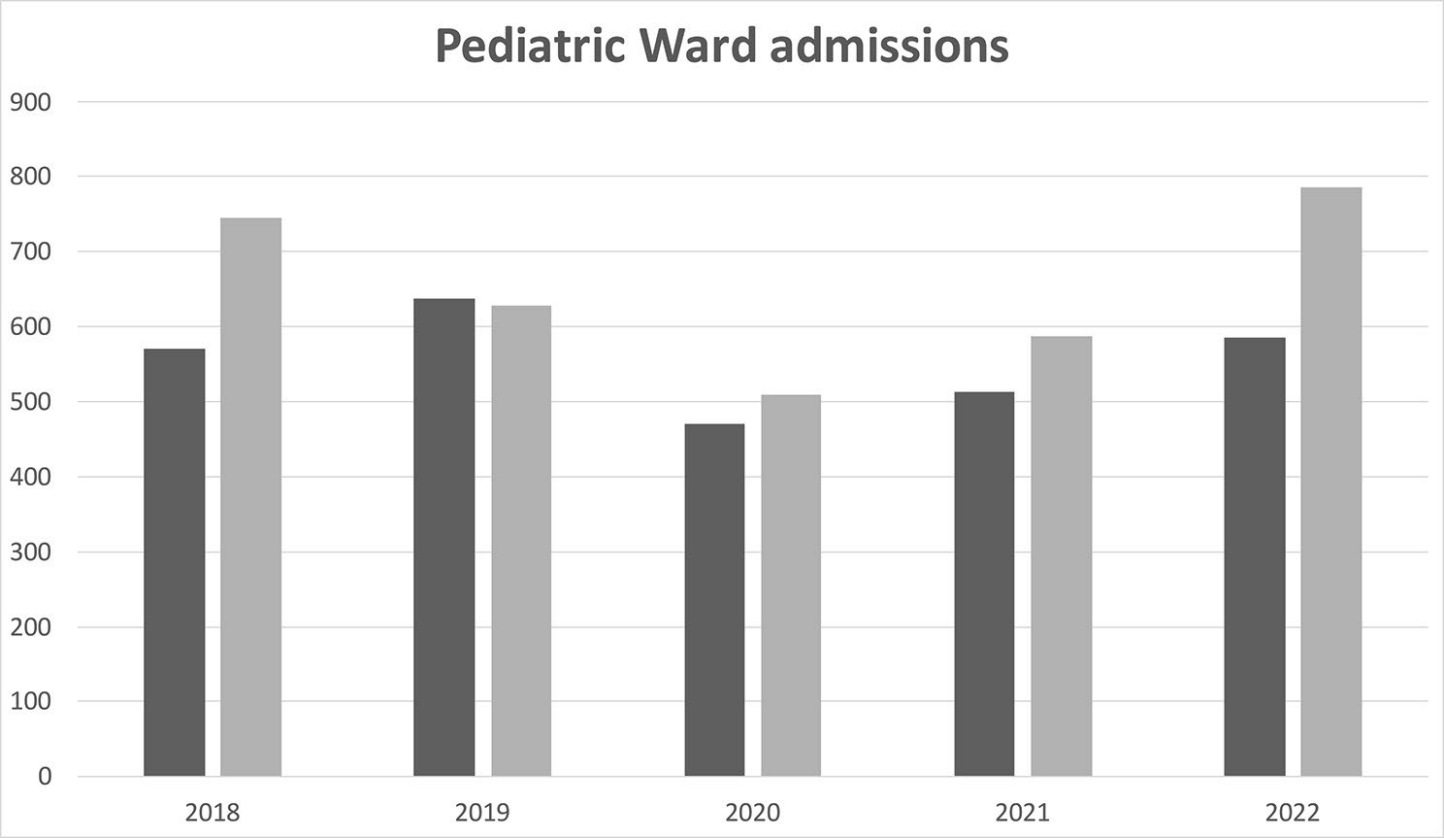
Volumes of accesses in the last few years at the Pediatric Ward. Dark and light grey bars represent ordinary hospitalizations and short-term observations (less than 36 h) respectively

The Pediatric Unit of Trento provides services for patients 0–14 years, mainly resident in the Province of Trento. Pediatric care is delivered in different settings: Ambulatory outpatient (No. 7402 accesses in 2022), Pediatric Ward (No. 1371), Pediatric Emergency Room (No. 17.610), Rare Disease Center (No. 224 patients in follow up, 388 visits) and Day Hospital Service (No. 201). In Figs. 1 and 2 we report the Emergency room and Pediatric Ward volumes of accesses in the last few years. The Pediatric Unit manages diseases in these subspecialties: general pediatrics, pediatric pulmonary and allergology, cardiology and vascular anomalies; pediatric endocrinology, obesity, skeletal diseases, diabetology, inherited metabolic disorders, gastroenterology, nephrology, rheumatology, onco-hematology, rare diseases and palliative care.

### Data source

We collected data on consumption and spending in the last two years of the COVID-19 pandemic (between January 2018 and December 2022). The source of economic data was the Planning and management control Service of APSS.

We considered as consumption and costs related to the COVID-19 pandemic the following: (1) antigen-based rapid diagnostic tests for COVID-19 used to screen for infectious individuals with symptoms suggestive of SARS-COV2 infection; (2) personal protective equipment such as head cap, face masks, gloves, visors and suits. We could not distinguish between sensors for oxygen saturation measurements, electrodes and infusion pump lines used during admissions due to COVID-19 or to other causes. We did not use a staff dedicated exclusively to perform screening tests for COVID-19 infection.

We conducted focused analyses of medical devices and drug categories with a major impact in relation to the clinic expenditure.

### Statistical analysis

We compared total spending of each pediatric setting, among the different years and subclasses, using ANOVA test.

## Results

The total spending of our Pediatric Unit from 2018 to 2022 was not statistically different (p = 0.99), but from 2018 to 2020 there was an increased spending for biologic drugs infused at the Day Hospital and at the Rare disease center. In Table 1 we report the allocation of the healthcare spending across the Healthcare services of our Pediatric Unit. This budget includes the costs for medical devices, drugs, and other health care product, while health care professionists’ fees, costs for laboratory and radiology services are not included. The decrease of the spending in the Day hospital, along with the increase at the Rare disease center, was due to the fact that the cost of a biologic drug infusion in a patient from 2020 to 2022 was attributed to the Rare disease center.

In the same period, the spending increased also in the outpatient clinic due to the introduction of new diabetes technologies.

In Table 2 we report the subdivision of the entire budget of the Pediatric Unit according to some macro-categories of expenditure: COVID-19, biologic drugs and devices related to diabetes management. The decrease in the spending of biologic drugs after 2020 is attributable to the death of a patient. Comparing all costs and the ones related to macro-categories of expenditure, between 2021 and 2022 there were no statistical differences. In Table 3 we report details of the spending, considering the same sub-group analysis with percentages referred to the budget of each Service of the Pediatric Unit.

**Table 1:** Allocation of the healthcare spending across Services of our Pediatric Unit

	% of total spending				
Health care service	2018	2019	2020	2021	2022
Emergency Room	3	4	5	8	11
Pediatric Ward	17	8	11	20	10
Day Hospital	56	49	27	14	6
Rare disease center	0	13	29	19	28
Outpatient Ambulatory	19	24	26	36	43
Direct drug distribution	4	2	2	2	2
**Total spend (euros)**	**2.190.500**	**2.470.584**	**2.483.931**	**2.141.220**	**2.147.529**

**Table 2:** COVID-19, biologic drugs and diabetes technology impact on the entire budget of the Pediatric Unit

Area of spending	2018 (euros)	%	2019 (euros)	%	2020 (euros)	%	2021 (euros)	%	2022 (euros)	%
COVID-19 related	-		-		126.834	5.1	129.633	6	172.040	8
Biologic drugs	1.420.598	64.8	1.550.807	62.8	1.342.464	54.0	891.367	35.4	704.892	32.8
Diabetes technology	381.559	17.4	570.417	23.1	617.543	24.9	759.023	41.6	888.287	41.3
Other	388.343	17.8	349.360	14.1	397.090	16.0	361.197	17.0	382.310	17.8
**Total spend (euros)**	**2.190.500**		**2.470.584**		**2.483.931**		**2.141.220**		**2.147.529**	

**Table 3:** Details of the spending, considering the most representative cost items

**2018**	**Emergency Room**	**Pediatric Ward**	**Day Hospital**	**Rare Disease center**	**Outpatient Ambulatory**	**Direct drug distribution**
Medical Devices	88.1%	16.7%	2.1%	-	96.6%	-
% diabetes technology	-	-	-	-	95.2%	-
% COVID-19 related	-	-	-	-	-	-
Drugs	8.9%	78.3%	97%	-	1.1%	99.9%
% biologic	-	64.9%	97.6%	-	-	85.8%
Other Health care product	2.9%	5%	0,9%	-	2.3%	-
**Total spending (euros)**	**76.001**	**363.043**	**1.230.248**	**-**	**414.781**	**93.244**
**2019**	**Emergency Room**	**Pediatric Ward**	**Day Hospital**	**Rare Disease center**	**Outpatient Ambulatory**	**Direct drug distribution**
Medical Devices	87.5%	41.3%	1.8%	-	97.6%	-
% diabetes technology	-	-	-	-	98.9%	-
% COVID-19 related	-	-	-	-	-	-
Drugs	9.7%	48.4%	97.8%	99.9%	0.9%	100%
% biologic	-	25.2%	98%	99.6%	-	83.1%
Other Health care product	2.8%	10.3%	0,4%	0.01%	1.5%	-
**Total spending (euros)**	**86.589**	**190.725**	**1.222.751**	**315.083**	**590.646**	**50.246**
**2020**	**Emergency Room**	**Pediatric Ward**	**Day Hospital**	**Rare Disease center**	**Outpatient Ambulatory**	**Direct drug distribution**
Medical Devices	93.1%	30.3%	6.9%	-	95.1%	-
% diabetes technology	-	-	-	-	99.9%	-
% COVID-19 related	51%	59%	3.5%	-		-
Drugs	3.7%	47.8%	88.9%	99.9%	1.4%	100%
% biologic	-	28.6%	95.6%	98.7%	-	80.2%
Other Health care product	3,2%	21.9%	4,2%	0.01%	3,5%	-
**Total spending (euros)**	**134.305**	**263.407**	**662.806**	**717.291**	**650.143**	**43.052**
**2021**	**Emergency Room**	**Pediatric Ward**	**Day Hospital**	**Rare Disease center**	**Outpatient Ambulatory**	**Direct drug distribution**
Medical Devices	83.3%	19.6%	8.1%	-	97.7%	-
% diabetes technology	-	-	-	-	97%	-
% COVID-19 related	54%	18%	3%	-	-	-
Drugs	3.7%	65.4%	86.3%	99.8%	1.4%	100%
% biologic	-	57%	74%	95%	-	76%
Other Health care product	13%	7%	5,6%	0.02%	0.9%	-
**Total spending (euros)**	**166.473**	**423.418**	**308.815**	**417.077**	**780.992**	**44.465**
**2022**	**Emergency Room**	**Pediatric Ward**	**Day Hospital**	**Rare Disease center**	**Outpatient Ambulatory**	**Direct drug distribution**
Medical Devices	91%	33.7%	12%	-	97%	-
% diabetes technology	-	-	-	-	97%	-
% COVID-19 related	68%	22%	2%	-	-	-
Drugs	3.1%	44%	74.3%	99.9%	1.6%	100%
% biologic	-	21%	65%	98%	-	52%
Other Health care product	5.9%	22.3%	13,7%	0.01%	1.4%	-
**Total spending (euros)**	**240.144**	**216.583**	**125.424**	**599.889**	**917.986**	**47.503**

### COVID-19 spending

COVID-19 spending accounted for 5.1 to 8% of the entire budget of the Pediatric Unit from 2020 to 2022.

In the Emergency room most of the spendings were related to medical devices and in our service costs for treatment of diseases related to pediatric surgery and orthopedics are included (8–12% of the spending). COVID-19 related costs accounted for 51–68% of the Emergency room budget for devices, in the period 2020–2022, and from 2021 mostly were due to COVID-19 antigen tests and less to individual protection equipment (Table 4). The spending in antigen tests increased from 2020 to 2022 because we stopped the use of molecular tests (that were accounted on a central hospital budget code) and we used only second-generation antigen tests, that costed from 14 to 15 euro for single test. Moreover, the number of accesses to the pediatric emergency room increased (Fig. 1) and our protocols still today (April 2023) indicate to screen children with symptoms related to SARS-COV2 infection. In 2021 and 2022 we performed 7.330 and 10.605 antigen tests, respectively. In the Pediatric ward and in other pediatric services the spending for these devices was less represented (2–22%), with the exception of a peak of 59% in the Pediatric ward in 2020.

**Table 4:** Emergency room spending: 54–68% was related to COVID-19 and details are presented

	2020 (euros)	2021 (euros)	2022 (euros)
**Antigen Test**	3.490	63.190	148.125
**Gloves, Head cap**	4.915	9.632	8.129
**Mask and suits, visors**	53.242	17.094	6.672
Other medical devices	62.370	67.898	65.937
Drugs	5.337	6.168	7.395
Other Health products	4.950	2.491	3.886
Total (euros)	134.304	166.473	240.144

### Biologic drugs

The total cost of the biologic drugs was 35.4% of the entire budget in 2021 and 41.3% in 2022. At the Pediatric Ward, Day hospital and Rare disease center, biologic drugs impacted on the budget with a mean percentage of 45%, 78% and 97% in the last three years, respectively. The costs were differently distributed between 2019 and 2022 due to infusion of the drug in one service or in another, but the number of patients treated was the same. The diseases treated were above all metabolic ones: No 1 patient with Gaucher disease, No 2 with Pompe disease, No 1 with Mucopolysaccaridosis type IV.Other biologics were used for inflammatory bowel diseases, juvenile arthritis and oncologic patients.

### Diabetes technology spending

T1D technology devices accounted for 41.6% of the entire budget in 2021 and 32.8% in 2022. On the outpatient clinic budget, these devices impacted with a percentage of 97% in 2021 and 97% in 2022. The costs were mostly due to flash and real time continuous glucose monitoring (CGM) (66–67%) because the number of patients who used this technology increased in the last years (No. 267). We calculated the cost of diabetes technology according to the different modality of treatment, and we found that at our Center the mean annual spending for patients was as follows: basal bolus insulin regimen: 230 euros; flash glucose monitoring: 950 euros; real-time CGM: 1.300-7.065; patch pump 4.500-5.000 euros; on traditional continuous subcutaneous insulin infusion (CSII): 3800–5000 euros (plus 184 euros of insulin); artificial pancreas system: 11.000 to 13.000 euros.

## Discussion

The analysis of the annual economic spending of our Pediatric Unit from 2018 to 2022 led to these findings:

1. The main impact on the budget was not due to COVID-19 pandemic related costs (5–8% of the total budget). The spending for COVID-19 antigen tests and personal protective equipment ranged from 51 to 68% of the Emergency room budget to 3–22% in other settings. The median cost per patient during the pandemic period has been previously reported as slightly higher in 2020 compared to 2019 [7] and even if the Authors did not report the detail of the spendings, probably the increase was mostly due to antigen test as we reported. We are still wondering if the epidemiological trend of SARS-COV-2 infection could allow a change in our protocols of patients’ screening.
2. We found that in our Province a small but growing population of children with inherited metabolic disorders, accounts for a high proportion (32.8% in 2022) of a Pediatric Unit spending; other studies reported that patients with medical care complexity (CMC) who comprised only 6% of the pediatric population, accounted for ∼40% of pediatric health care spending [15, 16] and treating one extra patient can change the budget of the entire Pediatric Unit. Biologic medicines are often used to treat many severe and life-threatening diseases, they have clinically proven efficacy, detailed indications for specific diseases and specific dosages. However, their use is associated to higher costs compared with traditional drugs, and pricing and reimbursement is controlled in Italy by an appropriate Agency (Agenzia Italiana del Farmaco - AIFA). The advent of biosimilars as well as of generic drugs into the market changed the costs of the therapy [17]. In our health care services, cost savings has been achieved in the pediatric area in the period 2021–2022 by the introduction of the generic drug of carglumic acid, and biosimilars of insulin glargine and somatropin agents; we are monitoring the market for biosimilars in inherited metabolic diseases (IMDs).
3. We found that among chronic diseases we face in the Outpatient Clinic, the costs for devices used to manage T1D account for 41.6% of the budget in the last years. The spending was mainly related to flash and real-time glucose monitoring, and we calculated the entire cost of technology in patients with T1D. We found that in 2021–2022 the annual cost of a patient treated with multiple daily injections (MDI) was 230 euros, with CSII 3800–5000 euros, with AP 11.000–13.000 euros. In literature is reported that T1D is associated with substantial economic burden but annual direct costs are due for the largest part to long-term diabetes complications, and not to technology use [18]. We have invested in technology to prevent future development of complications, trying to achieve optimal glucose control and improve patient related outcomes [19–22]. The cost of technology in adults with T1D in Italy, has been previously evaluated by Franciosi M. et al. in 2011 [23]: the Authors found that the cost of the treatment with MDI was 872 euros, while with CSII was 6000 euros, and cost-effectiveness analysis considered these technologies as cost effective [24]. The spending for technology is in line with our findings, considering the price increase due to inflation and to technological upgrade, while we saved on the cost of insulin through regional calls and the use of biosimilars.

To reduce the economic burden of diabetes technology, our Health care service company (APSS) put instruments with similar characteristics in competition on price (we included in the results also the costs of Free Style Libre 3® and Dexcom One®), and joins inter-regional calls for expression of interest in the area of diabetes medical devices.

Strengths of this study are: we analyzed real world data of accesses and spending of secondary level Pediatric Unit Health services and these data were not available in literature, to perform a benchmarking; our Province has only one public health care company and one administrative service, therefore the data are complete.

Limitations are: the results of the study could be not bench-market to tertiary care Pediatric Units that face high level specialty (as heart or brain surgery, dialysis, bone marrow transplant etc.) and the costs of devices can impact in a different way, but our data could be useful for Pediatric Units with a mission and performance volumes similar to ours; our spending budget does not include the costs for laboratory and radiology services and we are implementing the “SAP software” to collect this data in the future. Moreover, regional differences could be marked in Italy and in Europe, as direct and indirect costs depend on the flows of care needs and severity, as well as on clinical and administrative management.

## Conclusions

The impact of SARS-COV 2 on our Pediatric Unit hospital health care services accounts for only 5 to 9% of the spending, while the costs of pharmacologic biologic treatments as well as the spending for technology used in T1D management had a major impact on the budget. Cost savings could be achieved through generic and biosimilars introduction and with inter-regionals calls for technological devices; we emphasize how the control of spending in pediatric hospital care has moved from the bedside (savings on traditional drugs as antibiotics) to the bench of national or inter-regional round tables, to obtain discounts on the costs of biologic drugs and medical devices.

## Data Availability

The datasets used and/or analyzed during the current study are available from Roberto Franceschi on reasonable request.

## List of Abbreviations

APSS: Azienda Provinciale per i Servizi Sanitari
CGM: Continuous glucose monitoring
MDI: Multiple daily injections
CSII: Continuous subcutaneous insulin infusion
T1D: Type 1 diabetes
IMDs: Inherited metabolic diseases
